# Activin A is increased in the nucleus accumbens following a cocaine binge

**DOI:** 10.1038/srep43658

**Published:** 2017-03-08

**Authors:** Zi-Jun Wang, Jennifer A. Martin, Amy M. Gancarz, Danielle N. Adank, Fraser J. Sim, David M. Dietz

**Affiliations:** 1 Department of Pharmacology and Toxicology, Research Institute on Addictions, Program in Neuroscience, State University of New York at Buffalo, Buffalo, NY, USA; 2 Department of Psychology, State University of New York at Buffalo, Buffalo, NY, USA; 3 Department of Psychology, California State University Bakersfield, Bakersfield, CA, USA

## Abstract

Drug addiction is a long-lasting disease characterized by compulsive drug intake mediated in part by neuronal and biological adaptations in key brain areas, such as the nucleus accumbens (NAc). While we previously demonstrated involvement of the activin 2a receptor in drug taking, the role of its ligand, activin A, in cocaine relapse is unknown. Activin A levels in the NAc were assessed via ELISA and immunohistochemistry (in neurons, astrocytes, and microglia) following a cocaine binge paradigm. Cocaine exposure significantly increased the levels of activin A in the NAc of animals that had self-administered cocaine prior to the 14-day withdrawal compared with levels in saline controls. This was accompanied by an increase in the proportion of IBA1^+^ microglia in the NAc that were immunopositive for activin A. In contrast, the proportions of NeuN^+^ neurons and GFAP^+^ astrocytes that were immunopositive for activin A remained unaltered. In conclusion, these data suggest that increased secretion of activin A, particularly from microglia, in the NAc represents a novel potential target for the treatment of cocaine relapse.

Drug addiction is a chronic disease characterized by compulsive drug seeking and episodes of relapse despite prolonged periods of drug withdrawal^1^. The critical aspect of this disease is the transition from controlled drug use to uncontrolled addiction, which involves a gradual escalation of drug intake^2^. Relapse after extended periods of withdrawal occurs in an estimated 90% of cocaine addicts^3^,^4^, and remains a serious obstacle for effective treatment. In fact, there are currently no effective treatments for cocaine addiction, despite intense efforts to understand the underlying neurobiological mechanisms.

During chronic cocaine exposure or re-exposure following withdrawal, there are neuronal changes that occur in the nucleus accumbens (NAc)^5^,^6^. Such adaptations are thought to underlie increased drug seeking and craving, as well as the resistance to negative consequences, following extended periods of withdrawal^7^,^8^,^9^. There is also accruing evidence that glia play a role in mediating synaptic alterations associated with drug addiction. For example, microglia-derived IL-10 and TNF-α cytokines are involved in morphine conditioned place preference^10^,^11^,^12^,^13^,^14^ and cocaine sensitization^15^, respectively. In addition, microglia and astrocytes are involved with synaptic regulation via release of various neurotransmitters^16^,^17^,^18^,^19^, which may be important for neuronal plasticity in response to drugs of abuse. For example, cocaine self-administration and extinction training activates NAc astrocytes and reduces their colocalization with synapsin I, thus potentially altering synaptic strength after the cessation of chronic cocaine use^20^. Release or secretion of other factors may, therefore, also play a role in neurobiological changes in response to drug exposure and withdrawal.

We previously found that the TGF-β type 1 and activin 2a receptors are upregulated following cocaine self-administration^21^,^22^. The TGF-β superfamily ligand activin A is known to play important roles in regulating cell proliferation, differentiation, apoptosis, immune response, and endocrine function. The βA subunit composing activin A dimeric polypeptides is highly expressed in the brain and involved in CNS regeneration^16^. Although recent studies have examined its role in memory^23^ and anxiety behavior^24^, the role of activin A in cocaine relapse has not been determined.

The aim of this study was to determine whether activin A in the NAc is involved in drug addiction by using a rodent model of relapse. Compulsive cocaine-intake behaviors, which are a key component of drug addiction, can be modeled with an extended-access self-administration paradigm^2^. In addition, a cocaine binge test after prolonged withdrawal is useful for studying the neurobiological consequences of loss-of-control drug use^25^ and also has high face validity by representing the most common self-administration binge pattern after prolonged withdrawal in cocaine addicts. Thus, we utilized a cocaine binge test following prolonged withdrawal (14 days) from extended-access cocaine self-administration to investigate changes in activin A within the NAc.

## Results

### Increased activin A in the NAc after a cocaine binge

**Figure 1:**
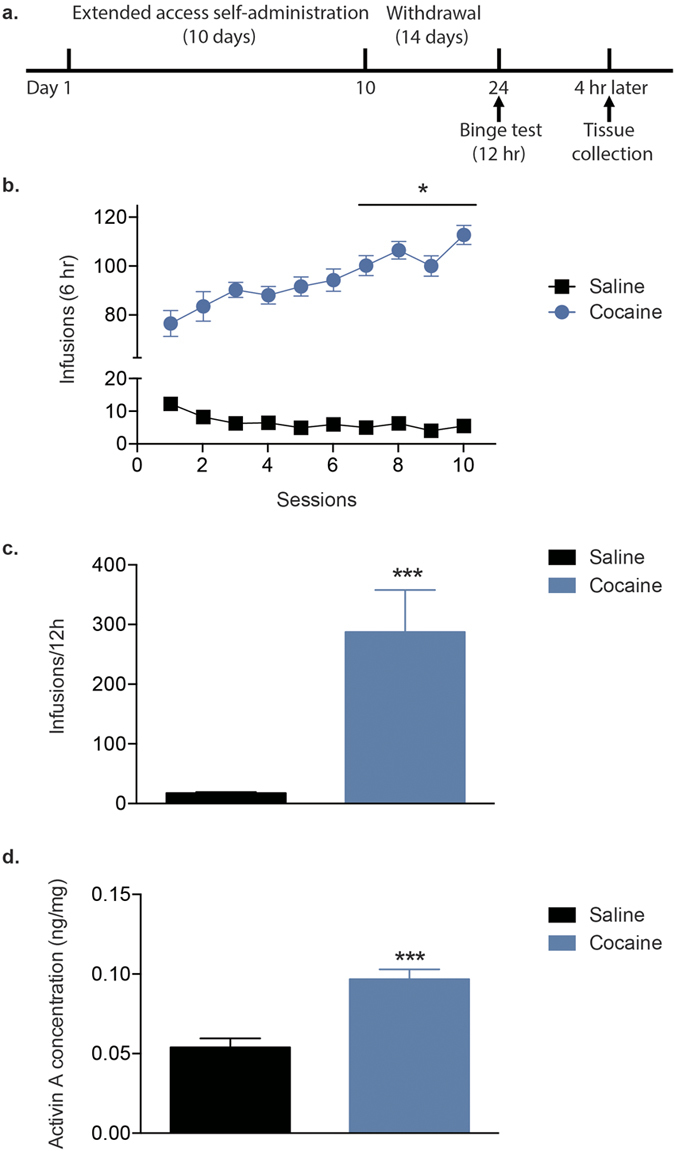
Cocaine binging increases the levels of activin A following a 14-d withdrawal from extended-access cocaine self-administration. (**a**) Timeline of behavioral testing and tissue collection. (**b**) The number of infusions earned during 10 d of extended-access cocaine (0.5 mg/kg/inf) or saline self-administration (*n* = 10/group). (**c**) Average number of infusions during the 12-h binge test. (**d**) Activin A concentration in the NAc after the cocaine binge (*n* = 6/group). Data are expressed as mean ± SEM, *P < 0.05, ***P < 0.001 *vs* saline.

To examine if activin A is regulated following relapse, rats underwent extended-access cocaine or saline self-administration for 10 d. A binge test was performed following a 14-d withdrawal to mimic the uncontrolled intake during a relapse episode, and animals were sacrificed 4 h later (Fig. 1a). A two-factor repeated measures ANOVA with drug (cocaine, saline; *n* = 10/group) as between-subjects factor and session as within-subject factor revealed main effects of drug (F_1,180_ = 3256, P < 0.001) and session (F_9,180_ = 4.275, P < 0.001) and an interaction between drug and session (F_9,180_ = 6.020, P < 0.001; Fig. 1b), indicating that animals assigned to cocaine groups self-administered significantly more infusions than rats assigned to saline groups. In addition, a Bonferroni post-hoc test revealed that the numbers of infusions in the cocaine group during the last four sessions were significantly increased compared with the first session (P < 0.05). As expected, rats self-administered significantly more infusions of cocaine compared with saline during the 12-h binge (t_18_ = 5.448, P < 0.001, *n* = 10/group; Fig. 1c). Importantly, levels of activin A in the NAc were significantly increased in the cocaine group compared with the saline control animals (t_10_ = 5.144, P < 0.001, *n* = 6/group; Fig. 1d). In addition, activin A levels did not differ between the saline group (0.053 ± 0.006 ng/mg) and the cocaine 14-day withdrawal without re-exposure to cocaine (0.054 ± 0.009 ng/mg, t_10_ = 0.0156, P = 0.987; behavioral data for this group not shown).

### Increased activin A from microglia in the NAc

**Figure 2:**
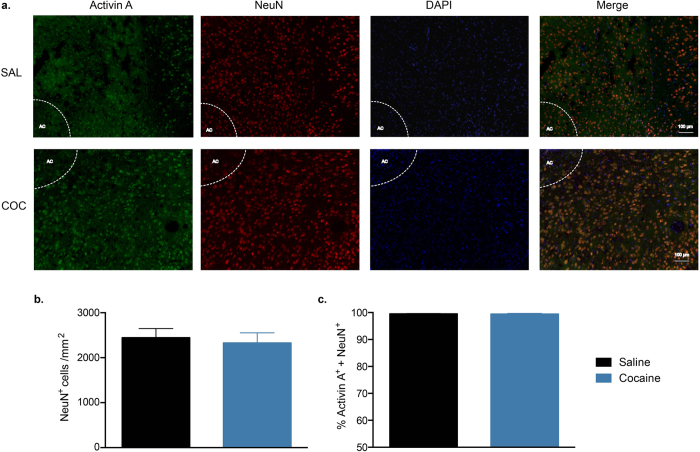
Double staining for activin A and the neuronal marker NeuN in the NAc. (**a**) Representative images of immunofluorescence staining in the NAc after cocaine (COC) or saline (SAL) self-administration during the binge test (AC: anterior commissure). (**b**) The number of NeuN^+^ cells in the NAc. (**c**) Percentage of NeuN^+^ cells that double-stained for activin A. Data are expressed as mean ± SEM, *n* = 4/group.

Previous reports have suggested that neurons are a large source of activin A in the CNS^26^. To determine if neurons were the source of increased activin A in the NAc following cocaine self-administration, we performed double staining for activin A and NeuN (neuron marker^27^, Fig. 2a). Cocaine exposure did not alter the number of NeuN^+^ cells (t_6_ = 0.390, P = 0.709, *n* = 4/group; Fig. 2b) or the percentage that were double-positive for activin A (t_6_ = 0.068, P = 0.947; Fig. 2c).

**Figure 3:**
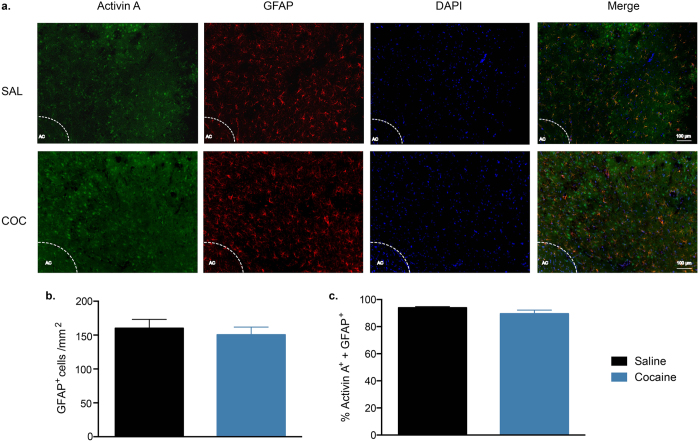
Double staining for activin A and the astrocyte marker GFAP in the NAc. (**a**) Representative images of immunofluorescence staining in the NAc after cocaine (COC) or saline (SAL) self-administration during the binge test (AC: anterior commissure). (**b**) The number of GFAP^+^ cells in the NAc. (**c**) Percentage of GFAP^+^ cells that double-stained for activin A. Data are expressed as mean ± SEM, *n* = 4/group.

Recent evidence indicates that glial cells also produce activin A^28^,^29^. Thus, activin A expression after cocaine relapse was examined in astrocytes and microglia. Double immunostaining for activin A and the astrocyte marker GFAP^20^; (Fig. 3a) showed that there was no difference in the number of GFAP^+^ cells (t_6_ = 0.560, P = 0.595, *n* = 4/group; Fig. 3b) or the percentage that were activin A^+^ (t_6_ = 1.615, P = 0.157; Fig. 3c).

**Figure 4:**
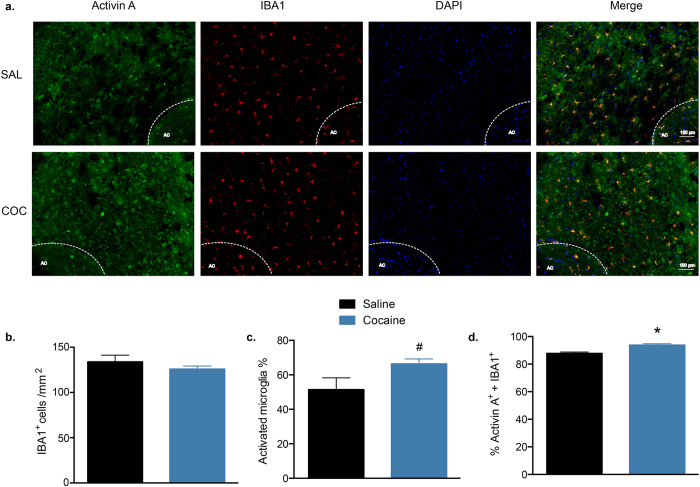
Double staining for activin A and the microglia marker IBA1 in the NAc. (**a**) Representative images of immunofluorescence staining in the NAc after cocaine (COC) or saline (SAL) self-administration during the binge test (AC: anterior commissure). (**b**) The number of IBA1^+^ cells in the NAc. (**c**) Percentage of IBA1^+^ cells exhibiting an activated morphology. (**d**) Percentage of IBA1^+^ cells that double-stained for activin A. Data are expressed as mean ± SEM, *P < 0.05, ^#^P < 0.1 *vs* saline, *n* = 4/group.

Finally, the microglial population in the NAc was examined after cocaine exposure by immunohistochemical staining for IBA1^30^ (Fig. 4a). There were no differences between the saline and cocaine groups in the numbers of IBA1^+^ cells (t_6_ = 0.990, P = 0.360, *n* = 4/group; Fig. 4b) or the percentages of activated microglia, as determined by the percentages of cells with an amoeboid morphology (t_6_ = 2.015, P = 0.090; Supplementary Fig. S1, Fig. 4c). Interestingly, the percentage of microglia that were immunopositive for activin A was significantly higher in the cocaine group (t_6_ = 4.379, P = 0.004; Fig. 4d). In addition, there was a significant positive correlation between the numbers of activated microglia and the numbers of activin A^+^ microglia (r = 0.767, P = 0.026, Supplementary Fig. S2). Taken together, these data indicate that the cocaine relapse-induced increase in activin A were more pronounced in microglia in comparison with levels found in neurons and astrocytes, and may be correlated with microglial activation.

## Discussion

In this study, we used extended-access cocaine self-administration followed by a binge test to study the neurobiology of cocaine relapse. After a 12-hour cocaine binge, there was a significant increase in activin A levels in the NAc. Although activin A was constitutively expressed within neuronal, astrocytic, and microglial cell populations, only microglia exhibited increase in expression. These results suggest that activin A, particularly from microglia, is a novel molecular adaption in the NAc that is associated with cocaine relapse behaviors.

Animal models that mimic certain aspects of cocaine addicts have been studied for years^31^. Compared to the two-hour short-access cocaine self-administration model, the extended-access model is considered as a better representative of compulsive and uncontrolled cocaine intake in the human condition^2^. In addition, it has been reported that both humans and rodents show an escalating-dose binge cocaine phenomenon^2^,^9^,^32^,^33^. Consistent with previous reports, our data show that during extended-access cocaine self-administration training, cocaine intake escalated compared to day 1, which may accelerate cocaine self-administration during the binge test and mimics relapse^34^.

The fact that activin A is altered during cocaine relapse is interesting, as it is involved not only in the modulation of cytokine networks in the brain^35^, but also in the regulation of other biological processes, such as memory formation^23^, anxiety behavior^24^, and alcohol-induced sedation^36^. Other cytokines, such as nuclear factor κB, regulate neuronal morphology in models of cocaine addiction^37^. Therefore, we speculate that increased activin A after cocaine binging may contribute to actin cycling to induce structural changes observed in the medium spiny neurons of the NAc, such as increases in dendritic spine density^38^. Indeed, we have shown that the activin 2a receptor signaling pathway is a necessary modulator of this cocaine-induced spine density, while also regulating drug-induced reinstatement^21^.

Microglia, as the major resident macrophage cells in the brain, have also been implicated in drug addiction. The psychostimulant methamphetamine induces activation of microglia in the brains of both rodents^39^ and humans^40^. Similarly, cocaine induces microglial activation in cocaine users^41^ and in the NAc^15^ and cerebellum^42^ of rats, as well as in cells cultured *in vitro*^43^. Our data show that after a cocaine binge, there is a significant increase in the proportion of activin A^+^ microglia. Interestingly, the numbers of activated microglia are positively correlated with the numbers of activin A^+^ microglia, indicating that microglia-derived activin A may be a target for the treatment of neurological and perhaps psychiatric diseases such as drug addiction^16^.

Previous reports indicate that cocaine self-administration and extinction results in a significant decrease in astrocyte number in the NAc core region^20^, whereas an acute cocaine injection following a three-week withdrawal from repeated daily cocaine injections results increases GFAP expression selectively in the NAc shell^44^. We did not observe any changes in astrocyte number or percentage of activin A^+^ GFAP^+^ double-stained cells after a cocaine binge. These discrepancies are likely due to differences in cocaine-administration paradigms (experimenter-administered *vs* self-administered) and withdrawal periods. Furthermore, our paradigm utilized a binge test following withdrawal from extended-access cocaine self-administration at 0.5 mg/kg/inf, which differs from cocaine re-exposure by i.p. injection after extinction training from short-access cocaine self-administration (2 hours/session) at dose of 0.2 mg/inf as used in the previous reports. Moreover, the subregions of the NAc have also been shown to play different roles in cocaine addiction^45^. Further study is needed to assess NAc subregion differences in the binge model.

Although microglia and astrocytes synthesize activin A, the primary source of activin A in the brain is thought to be neurons^28^,^29^. One limitation of our study is that we cannot exclude the possibility that the increased levels of activin A are from increased neuronal synthesis. We were unable to detect an increase in expression in this population, as nearly all of the NAc neurons were also activin A^+^, and immunohistochemistry is a largely qualitative, not quantitative, technique. Further studies are needed to address whether the increased activin A is solely from microglia, although this would not appear likely given the magnitude of increase in activin A levels following a binge. Additionally, while we demonstrate an increase in activin A^+^ IBA1^+^ microglia, a more complete and detailed analysis of microglial identification and activation following cocaine exposure will provide further insight into how non-neuronal cell types contribute to long-term behavioral adaptations to drugs of abuse.

In conclusion, we show that activin A is increased in the NAc after a cocaine binge following withdrawal from extended-access self-administration, and that this increase is likely from the resident microglia. These results expand upon the spectrum of neurobiological adaptions contributing to binging following prolonged withdrawal from chronic cocaine exposure. These data demonstrate that activin A is a potential target for intervention towards an effective pharmacotherapy in the treatment of cocaine relapse.

## Methods

### Animals

Male Sprague−Dawley rats (300 g; Harlan Laboratories, Indianapolis, IN, USA) were allowed to acclimate for 3 d upon arrival to the colony room, and housed on a 12-h light-dark cycle with *ad libitum* access to food and water. Rats were singly housed following surgery and for the duration of the experiment in order to protect the catheter/harness assembly. Testing took place 7 d/wk during the rats’ dark phase of the light-dark cycle. This study and all experimental protocols were approved by the Institutional Animal Care and Use Committee of the State University of New York at Buffalo, and were carried out in accordance with the guidelines of the National Institutes of Health Guide for the Care and Use of Laboratory Animals.

### Drugs

Solutions of (-)-cocaine hydrochloride (generously gifted through the NIDA drug supply program), dissolved in sterile 0.9% saline, were prepared on a weekly basis (acquisition, 4.5 mg/mL; extended access, 1.7 mg/mL; cocaine binge, 1.0 mg/mL). Pump durations were adjusted according to body weight on a daily basis in order to deliver the correct dose of drug for each individual.

### Self-administration test chambers

The experimental chambers have been described elsewhere^9^,^46^. Briefly, 16 standard test chambers (MED Associates, Inc., St. Albans, VT, USA) were equipped with two snout-poke holes that were monitored with infrared detectors. Two stimulus lights were mounted above the snout-poke holes, and a houselight was mounted in the middle of the back wall of the test chamber. All test chambers were housed in sound-attenuating chambers. Test chambers were computer controlled through a MED Associates interface with a temporal resolution of 0.01 s.

### Jugular catheterization and patency tests

Rats were implanted with chronic indwelling jugular catheters and allowed 7 d to recover after the surgery as previously described^21^,^47^. Catheters were flushed daily with 0.2 mL of a heparinized saline solution (50 IU/mL) containing enrofloxacin (4 mg/mL) to preserve patency. Tests of patency occurred once per week throughout the behavioral testing and during the 14-d abstinence from cocaine. Catheter patency was tested using an infusion of ketamine hydrochloride (0.5 mg/kg in 0.05 mL saline; i.v.) and behavioral responses were observed. Loss of muscle tone and righting reflexes served as behavioral indicators of patency. Only rats with patent catheters were used in data analyses. All self-administration procedures were performed based on previous studies^9^.

### Acquisition of self-administration

One week after jugular catheter surgery, rats were trained to self-administer cocaine (1.0 mg/kg/inf) or saline for 1 h each day. Responses to the active snout-poke hole resulted in infusions of drug followed by a 30-s time-out period using a fixed ratio (FR) 1 schedule of reinforcement, which was increased daily to an FR5 schedule. Following each session, catheters were flushed, and rats were returned to the colony room. The criterion for acquisition of cocaine self-administration was an average of ten infusions per day during the last 3 d of the acquisition period.

### Extended-access self-administration

Following self-administration acquisition, rats underwent a 10-d period of extended access to cocaine self-administration (0.5 mg/kg/inf). During this time, rats were allowed 6 h of access to cocaine (*n* = 10) or saline (*n* = 10) according to an FR5 schedule of reinforcement^2^,^9^,^38^. Following each session, catheters were flushed, and rats were returned to the colony room.

### Binge test

Following a 14-d withdrawal period, rats were tested during a single 12-h period during which responses to the active snout-poke hole resulted in infusions of cocaine (0.3 mg/kg/inf., for cocaine SA group) or saline (for saline SA group) according to an FR5 schedule of reinforcement (*n* = 10/group). Only rats that had reached criterion of acquisition and maintained catheter patency were used in this phase of the experiment. Failure to achieve acquisition or respond for cocaine resulted in removal of the animal from the remainder of the study.

### Tissue collection and ELISA

Four hours following the cocaine binge test, rats (*n* = 6/group) were sacrificed by rapid decapitation, brains were harvested and sliced into 1-mm thick sections using a brain matrix (Braintree Scientific, Inc., Braintree, MA, USA), and 2-mm diameter tissue punches from the NAc were collected and rapidly frozen on dry ice. NAc tissue punches were homogenized in buffer [0.32 M sucrose, 5 mM Tris-HCl (pH 8.0), protease inhibitor tablet (Hoffman-La Roche, AG, Basel, Switzerland)], and homogenates were centrifuged at 20,000 × *g* at 4 °C. Activin A levels were measured using an ELISA kit (Quantikine Activin Assay; R&D Systems, Inc., Minneapolis, MN, USA) according to the manufacturer’s instructions and as previously described^24^,^48^.

### Tissue preparation and immunostaining

To determine the cell-type specific expression levels of activin A in the NAc, we used immunofluorescent staining methods as previously described with some modifications^49^,^50^. Rats (*n* = 4/group) were sacrificed 4 h after the binge test via transcardial perfusion of PBS followed by 4% formaldehyde. Whole brains were immediately removed and post-fixed at 4 °C for 24 h and then immersed in 30% sucrose in 0.01 M PBS (pH 7.4) at 4 °C for cryoprotection. Coronal sections encompassing the NAc (+2.7 through + 0.7 mm from bregma, based on Paxinos & Watson^51^) were cryosectioned at a thickness of 20 μm, and every third section was collected. Brain sections were blocked in 3% normal donkey serum with 0.3% Triton-X for 2 h at 4 °C. Sections were then incubated overnight at room temperature in primary antibodies diluted in PBS with 3% normal donkey serum and 0.3% Tween-20: anti-activin A (1:40; Wako, Osaka, Japan) and either anti-NeuN (1:1000; Millipore, Billerica, MA, USA), anti-GFAP (1:1000; Millipore), or anti-IBA1 (1:1000; Wako). Alexa Fluor 488-conjugated and Cy3 AffiniPure secondary antibodies were used at 1:800 (Jackson ImmunoResearch Laboratories, Inc., West Grove, PA, USA). Colocalization was assessed via imaging on an Olympus IX51 microscope under 10× magnification. For each animal, 4–5 double-immunostained sections representing the anterior to posterior NAc were selected according to the brain atlas (+2.7 through + 0.7 mm from bregma, based on Paxinos & Watson^51^). The numbers of NeuN-, GFAP-, and IBA1-immunoreactive cells from bilateral NAc region (within the field of view at 10x magnification) were counted and averaged, and the percentages of cells that were also activin A immunopositive were determined. In addition, microglial morphology was assessed to quantify the percentages of activated microglia. Activation of microglia leads to a morphological change, which includes a shift from the ramified form (inactive) into a less ramified structure (activated), as observed in response to stimuli such as lipopolysaccharide or fractalkine^17^,^52^. All image acquisition and analyses were conducted by investigators blind to the experimental conditions.

### Data analysis

Two-factor repeated measures ANOVA was performed on the number of infusions during extended access, followed by Bonferroni post-hoc tests. Comparisons between two groups (saline *vs* cocaine) were performed using unpaired Student’s *t* tests. All data were analyzed using SPSS software (IBM Corp., Armonk, NY, USA), and are represented as the mean ± SEM, with P < 0.05 indicating significance.

## Additional Information

**How to cite this article:** Wang, Z.-J. *et al*. Activin A is increased in the Nucleus Accumbens following a cocaine binge. *Sci. Rep.* **7**, 43658; doi: 10.1038/srep43658 (2017).

**Publisher's note:** Springer Nature remains neutral with regard to jurisdictional claims in published maps and institutional affiliations.

## Supplementary Material

Supplementary Information

## References

[b1] Ridenour T. A., Maldonado-Molina M., Compton W. M., Spitznagel E. L. & Cottler L. B. Factors associated with the transition from abuse to dependence among substance abusers: implications for a measure of addictive liability. Drug and alcohol dependence 80, 1–14, doi: 10.1016/j.drugalcdep.2005.02.005 (2005). 16157227 PMC1435339

[b2] Ahmed S. H. & Koob G. F. Transition from moderate to excessive drug intake: change in hedonic set point. Science 282, 298–300 (1998). 9765157 10.1126/science.282.5387.298

[b3] DeJong W. Relapse prevention: an emerging technology for promoting long-term drug abstinence. The International journal of the addictions 29, 681–705 (1994). 8034380 10.3109/10826089409047904

[b4] O’Brien C. P. Treatment of alcoholism as a chronic disorder. Alcohol 11, 433–437 (1994). 7865139 10.1016/0741-8329(94)90063-9

[b5] Nestler E. J. Epigenetic mechanisms of drug addiction. Neuropharmacology 76 Pt B, 259–268, doi: 10.1016/j.neuropharm.2013.04.004 (2014). 23643695 PMC3766384

[b6] Russo S. J. . The addicted synapse: mechanisms of synaptic and structural plasticity in nucleus accumbens. Trends in neurosciences 33, 267–276, doi: 10.1016/j.tins.2010.02.002 (2010). 20207024 PMC2891948

[b7] Loweth J. A. . Synaptic depression via mGluR1 positive allosteric modulation suppresses cue-induced cocaine craving. Nature neuroscience 17, 73–80, doi: 10.1038/nn.3590 (2014). 24270186 PMC3971923

[b8] Loweth J. A., Tseng K. Y. & Wolf M. E. Adaptations in AMPA receptor transmission in the nucleus accumbens contributing to incubation of cocaine craving. Neuropharmacology 76 Pt B, 287–300, doi: 10.1016/j.neuropharm.2013.04.061 (2014). 23727437 PMC3836860

[b9] Gancarz-Kausch A. M., Adank D. N. & Dietz D. M. Prolonged withdrawal following cocaine self-administration increases resistance to punishment in a cocaine binge. Scientific reports 4, 6876, doi: 10.1038/srep06876 (2014). 25363133 PMC4217113

[b10] Schwarz J. M., Hutchinson M. R. & Bilbo S. D. Early-life experience decreases drug-induced reinstatement of morphine CPP in adulthood via microglial-specific epigenetic programming of anti-inflammatory IL-10 expression. The Journal of neuroscience : the official journal of the Society for Neuroscience 31, 17835–17847, doi: 10.1523/JNEUROSCI.3297-11.2011 (2011). 22159099 PMC3259856

[b11] Zhang X. Q. . Activation of p38 signaling in the microglia in the nucleus accumbens contributes to the acquisition and maintenance of morphine-induced conditioned place preference. Brain, behavior, and immunity 26, 318–325, doi: 10.1016/j.bbi.2011.09.017 (2012). 22004988

[b12] Hutchinson M. R. . Reduction of opioid withdrawal and potentiation of acute opioid analgesia by systemic AV411 (ibudilast). Brain, behavior, and immunity 23, 240–250, doi: 10.1016/j.bbi.2008.09.012 (2009). PMC2662518 18938237

[b13] Hutchinson M. R. . Minocycline suppresses morphine-induced respiratory depression, suppresses morphine-induced reward, and enhances systemic morphine-induced analgesia. Brain, behavior, and immunity 22, 1248–1256, doi: 10.1016/j.bbi.2008.07.008 (2008). PMC2783326 18706994

[b14] Schwarz J. M. & Bilbo S. D. Adolescent morphine exposure affects long-term microglial function and later-life relapse liability in a model of addiction. The Journal of neuroscience: the official journal of the Society for Neuroscience 33, 961–971, doi: 10.1523/JNEUROSCI.2516-12.2013 (2013). 23325235 PMC3713715

[b15] Lewitus G. M. . Microglial TNF-alpha Suppresses Cocaine-Induced Plasticity and Behavioral Sensitization. Neuron 90, 483–491, doi: 10.1016/j.neuron.2016.03.030 (2016). 27112496 PMC4860141

[b16] Miron V. E. . M2 microglia and macrophages drive oligodendrocyte differentiation during CNS remyelination. Nature neuroscience 16, 1211–1218, doi: 10.1038/nn.3469 (2013). 23872599 PMC3977045

[b17] Hanisch U. K. & Kettenmann H. Microglia: active sensor and versatile effector cells in the normal and pathologic brain. Nature neuroscience 10, 1387–1394, doi: 10.1038/nn1997 (2007). 17965659

[b18] Suzuki T. . Production and release of neuroprotective tumor necrosis factor by P2X7 receptor-activated microglia. The Journal of neuroscience: the official journal of the Society for Neuroscience 24, 1–7, doi: 10.1523/JNEUROSCI.3792-03.2004 (2004). 14715932 PMC6729576

[b19] Ashhad S. & Narayanan R. Active dendrites regulate the impact of gliotransmission on rat hippocampal pyramidal neurons. Proceedings of the National Academy of Sciences of the United States of America, doi: 10.1073/pnas.1522180113 (2016). PMC4988595 27217559

[b20] Scofield M. D. . Cocaine Self-Administration and Extinction Leads to Reduced Glial Fibrillary Acidic Protein Expression and Morphometric Features of Astrocytes in the Nucleus Accumbens Core. Biological psychiatry, doi: 10.1016/j.biopsych.2015.12.022 (2015). PMC4930433 26946381

[b21] Gancarz A. M. . Activin receptor signaling regulates cocaine-primed behavioral and morphological plasticity. Nature neuroscience 18, 959–961, doi: 10.1038/nn.4036 (2015). 26030849 PMC4599345

[b22] Gancarz-Kausch A. M. . Transforming growth factor beta receptor 1 is increased following abstinence from cocaine self-administration, but not cocaine sensitization. PloS one 8, e83834, doi: 10.1371/journal.pone.0083834 (2013). 24386286 PMC3875479

[b23] Ageta H. . Activin plays a key role in the maintenance of long-term memory and late-LTP. Learning & memory 17, 176–185, doi: 10.1101/lm.16659010 (2010). 20332189

[b24] Ageta H. . Activin in the brain modulates anxiety-related behavior and adult neurogenesis. PloS one 3, e1869, doi: 10.1371/journal.pone.0001869 (2008). 18382659 PMC2270335

[b25] Lynch W. J., Nicholson K. L., Dance M. E., Morgan R. W. & Foley P. L. Animal models of substance abuse and addiction: implications for science, animal welfare, and society. Comparative medicine 60, 177–188 (2010). 20579432 PMC2890392

[b26] Abdipranoto-Cowley A. . Activin A is essential for neurogenesis following neurodegeneration. Stem cells 27, 1330–1346, doi: 10.1002/stem.80 (2009). 19489097 PMC2733378

[b27] Wolf H. K. . NeuN: a useful neuronal marker for diagnostic histopathology. The journal of histochemistry and cytochemistry : official journal of the Histochemistry Society 44, 1167–1171 (1996). 8813082 10.1177/44.10.8813082

[b28] Jeong J., Ahn M., Sim K. B., Moon C. & Shin T. Immunohistochemical analysis of activin A expression in spinal cords of rats with clip compression injuries. Acta histochemica 116, 747–752, doi: 10.1016/j.acthis.2014.01.002 (2014). 24529943

[b29] Wilms H. . Regulation of activin A synthesis in microglial cells: pathophysiological implications for bacterial meningitis. Journal of neuroscience research 88, 16–23, doi: 10.1002/jnr.22185 (2010). 19681171

[b30] Garcia A. D., Doan N. B., Imura T., Bush T. G. & Sofroniew M. V. GFAP-expressing progenitors are the principal source of constitutive neurogenesis in adult mouse forebrain. Nature neuroscience 7, 1233–1241, doi: 10.1038/nn1340 (2004). 15494728

[b31] Haney M. & Spealman R. Controversies in translational research: drug self-administration. Psychopharmacology 199, 403–419, doi: 10.1007/s00213-008-1079-x (2008). 18283437 PMC2731701

[b32] Mantsch J. R., Yuferov V., Mathieu-Kia A. M., Ho A. & Kreek M. J. Effects of extended access to high versus low cocaine doses on self-administration, cocaine-induced reinstatement and brain mRNA levels in rats. Psychopharmacology 175, 26–36, doi: 10.1007/s00213-004-1778-x (2004). 15042275

[b33] Picetti R., Ho A., Butelman E. R. & Kreek M. J. Dose preference and dose escalation in extended-access cocaine self-administration in Fischer and Lewis rats. Psychopharmacology 211, 313–323, doi: 10.1007/s00213-010-1899-3 (2010). 20559822 PMC2926930

[b34] Quadros I. M. & Miczek K. A. Two modes of intense cocaine bingeing: increased persistence after social defeat stress and increased rate of intake due to extended access conditions in rats. Psychopharmacology 206, 109–120, doi: 10.1007/s00213-009-1584-6 (2009). 19513697 PMC4371736

[b35] Robson N. C. . Activin-A: a novel dendritic cell-derived cytokine that potently attenuates CD40 ligand-specific cytokine and chemokine production. Blood 111, 2733–2743, doi: 10.1182/blood-2007-03-080994 (2008). 18156495

[b36] Zheng F. . Activin Controls Ethanol Potentiation of Inhibitory Synaptic Transmission Through GABAA Receptors and Concomitant Behavioral Sedation. Neuropsychopharmacology: official publication of the American College of Neuropsychopharmacology 41, 2024–2033, doi: 10.1038/npp.2015.372 (2016). 26717882 PMC4908639

[b37] Russo S. J. . Nuclear factor kappa B signaling regulates neuronal morphology and cocaine reward. The Journal of neuroscience: the official journal of the Society for Neuroscience 29, 3529–3537, doi: 10.1523/JNEUROSCI.6173-08.2009 (2009). 19295158 PMC2677656

[b38] Cahill M. E. . Bidirectional Synaptic Structural Plasticity after Chronic Cocaine Administration Occurs through Rap1 Small GTPase Signaling. Neuron 89, 566–582, doi: 10.1016/j.neuron.2016.01.031 (2016). 26844834 PMC4743039

[b39] Thomas D. M., Walker P. D., Benjamins J. A., Geddes T. J. & Kuhn D. M. Methamphetamine neurotoxicity in dopamine nerve endings of the striatum is associated with microglial activation. The Journal of pharmacology and experimental therapeutics 311, 1–7, doi: 10.1124/jpet.104.070961 (2004). 15163680

[b40] Sekine Y. . Methamphetamine causes microglial activation in the brains of human abusers. The Journal of neuroscience : the official journal of the Society for Neuroscience 28, 5756–5761, doi: 10.1523/JNEUROSCI.1179-08.2008 (2008). 18509037 PMC2491906

[b41] Little K. Y. . Decreased brain dopamine cell numbers in human cocaine users. Psychiatry research 168, 173–180, doi: 10.1016/j.psychres.2008.10.034 (2009). 19233481

[b42] Lopez-Pedrajas R. . Cocaine promotes oxidative stress and microglial-macrophage activation in rat cerebellum. Frontiers in cellular neuroscience 9, 279, doi: 10.3389/fncel.2015.00279 (2015). 26283916 PMC4516895

[b43] Liao K. . Cocaine-mediated induction of microglial activation involves the ER stress-TLR2 axis. Journal of neuroinflammation 13, 33, doi: 10.1186/s12974-016-0501-2 (2016). 26860188 PMC4748483

[b44] Bowers M. S. & Kalivas P. W. Forebrain astroglial plasticity is induced following withdrawal from repeated cocaine administration. The European journal of neuroscience 17, 1273–1278 (2003). 12670315 10.1046/j.1460-9568.2003.02537.x

[b45] Ito R., Robbins T. W. & Everitt B. J. Differential control over cocaine-seeking behavior by nucleus accumbens core and shell. Nature neuroscience 7, 389–397, doi: 10.1038/nn1217 (2004). 15034590

[b46] Sun H. . BAZ1B in Nucleus Accumbens Regulates Reward-Related Behaviors in Response to Distinct Emotional Stimuli. The Journal of neuroscience: the official journal of the Society for Neuroscience 36, 3954–3961, doi: 10.1523/JNEUROSCI.3254-15.2016 (2016). 27053203 PMC4821908

[b47] Wang Z. J. . BRG1 in the Nucleus Accumbens Regulates Cocaine-Seeking Behavior. Biological psychiatry in press (2016). 10.1016/j.biopsych.2016.04.020 PMC5050080 27422367

[b48] Hasegawa Y. . Acute modulation of synaptic plasticity of pyramidal neurons by activin in adult hippocampus. Frontiers in neural circuits 8, 56, doi: 10.3389/fncir.2014.00056 (2014). 24917791 PMC4040441

[b49] Wang Z. J. . Glucocorticoid receptors in the locus coeruleus mediate sleep disorders caused by repeated corticosterone treatment. Scientific reports 5, 9442, doi: 10.1038/srep09442 (2015). 25801728 PMC4371174

[b50] Abiraman K. . Anti-muscarinic adjunct therapy accelerates functional human oligodendrocyte repair. The Journal of neuroscience: the official journal of the Society for Neuroscience 35, 3676–3688, doi: 10.1523/JNEUROSCI.3510-14.2015 (2015). 25716865 PMC4464275

[b51] Paxinos G. & Watson C. The Rat Brain In Stereotaxic Coordinates. (Elselvier, 2009). 10.1016/0165-0270(80)90021-7 6110810

[b52] Torres-Platas S. G. . Morphometric characterization of microglial phenotypes in human cerebral cortex. Journal of neuroinflammation 11, 12, doi: 10.1186/1742-2094-11-12 (2014). 24447857 PMC3906907

