# Supplementary material for: Activin A is increased in the nucleus accumbens following a cocaine binge
Source: Sci Rep. 2017 Mar 8;7:43658. doi: 10.1038/srep43658 (PMC5341561; doi:10.1038/srep43658)
Supplement: Supplementary Information [file srep43658-s1.pdf]

Activin A is increased in the Nucleus Accumbens following a cocaine binge

Supplemental Information

Zi-Jun Wang[a,b], Jennifer A. Martin[a,b], Amy M. Gancarz[a,b,c], Danielle N. Adank[a], Fraser J. Sim[a], David M. Dietz[a,b,*]

[a]Department of Pharmacology and Toxicology, Research Institute on Addictions, Program in Neuroscience, State University of New York at Buffalo, Buffalo, NY; [b]Department of Psychology, State University of New York at Buffalo, Buffalo, NY; [c]Department of Psychology, California State University Bakersfield, Bakersfield, CA

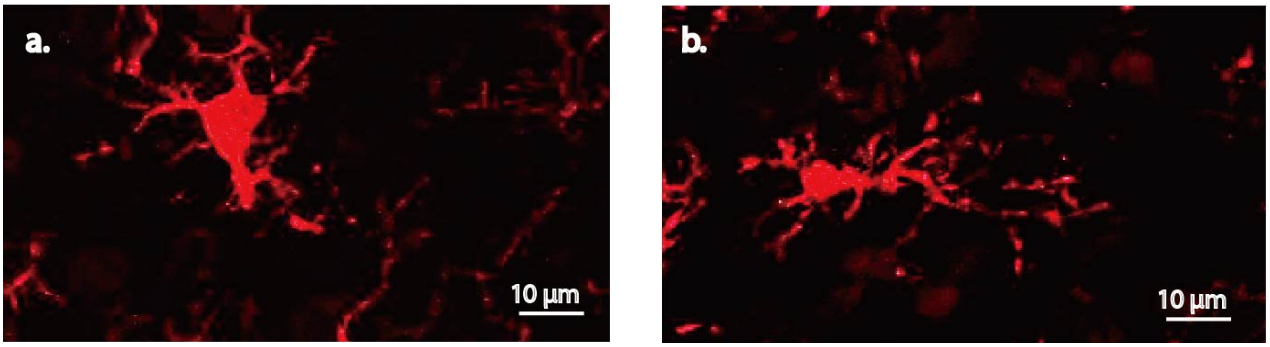

Figure S1. IBA1-immunohistochemistry revealing microglial cells with amoeboid (a, activated) and ramified (b, non-activated) morphology.

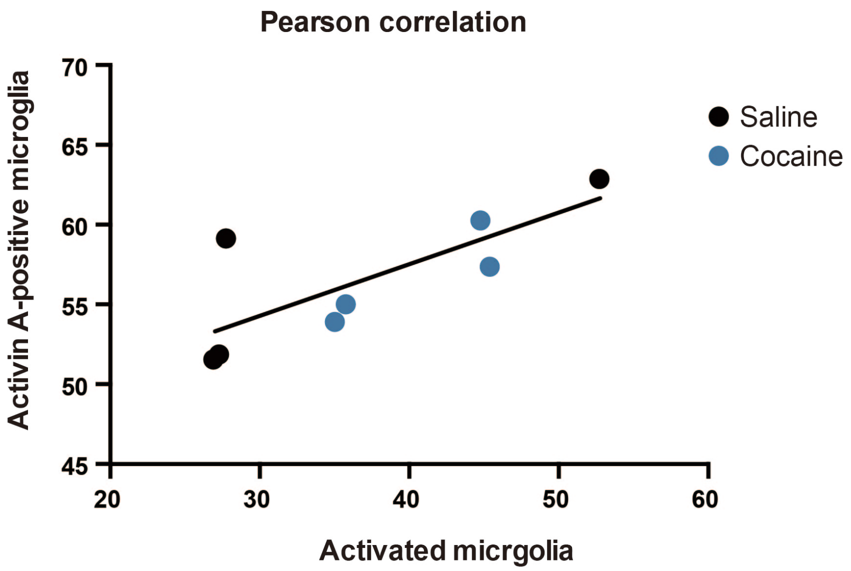

Figure S2. Correlation between activated microglia and activin A$^+$ microglia. Pearson correlation revealed that there is a significant positive correlation between activated microglia number and activin A$^+$ microglia number ($r = 0.767$, $P = 0.026$).
